# Editorial: Advances in understanding and treating post-traumatic stress disorder

**DOI:** 10.3389/fnhum.2025.1599415

**Published:** 2025-05-06

**Authors:** Jenny J. W. Liu, Nasser Kashou

**Affiliations:** ^1^Schulich School of Medicine and Dentistry, Western University, London, ON, Canada; ^2^The Ohio State University, Columbus, OH, United States

**Keywords:** post-traumatic stress disorder (PTSD), measurement based care, personalized and precision medicine (PPM), treatment, mental health

Post-Traumatic Stress Disorder (PTSD) remains one of the most challenging mental health conditions, presenting clinicians, researchers, and patients with diverse symptom profiles and varied clinical outcomes. This Research Topic highlights advancements underscoring an emerging shift toward systematically collecting patient-specific data to tailor and adapt treatment strategies, addressing persistent challenges such as treatment resistance, symptom variability, and limited individualized care.

Amid global challenges, including humanitarian crises and shifting healthcare priorities, addressing PTSD has become increasingly urgent. Rising trauma exposure among military personnel, first responders, and civilian populations emphasizes the need to invest in innovative, accessible, and effective treatments. Economic pressures and resource limitations across healthcare systems globally further underline the importance of cost-effective solutions and international collaboration to enhance accessibility for diverse patient populations.

Macy et al. highlight the potential of digital therapeutics, specifically heart rate variability biofeedback (HRV-BFB), to correct autonomic dysfunction central to PTSD pathology. Beyond symptom management, HRV-BFB may significantly reduce stigma and enhance health literacy, potentially improving patient engagement, adherence, and supported self-management. Nevertheless, widespread adoption faces regulatory challenges, inconsistent funding, and fragmented healthcare infrastructures, emphasizing the need for international collaboration and evidence-informed policy advocacy to support broader implementation.

Guo et al. delve into fear memory erasure, distinguishing it from traditional extinction processes, which often fail due to spontaneous recovery, reinstatement, and renewal of fear memories. Their detailed neuroscientific insights highlight the potential for precise neurobiological interventions tailored to individual neural profiles, but also emphasize complex ethical and practical considerations inherent in permanently modifying traumatic memories. Navigating these challenges will require cautious ethical deliberation, clear regulatory guidance, and rigorous scientific exploration.

Shannon and Geller discuss MDMA (3,4-methylenedioxymethamphetamine) -assisted psychotherapy, highlighting significant regulatory advancements, notably current FDA consideration, as marking a transformative integration of pharmacological and psychotherapeutic modalities. While MDMA itself is not new, its regulatory progression signals a critical shift toward integrated mental healthcare, potentially benefiting diverse populations beyond PTSD, including those with anxiety, addiction, and marginalized groups traditionally underserved by conventional psychiatric interventions. However, global regulatory approval remains cautious, reflecting ongoing societal and healthcare policy debates that must balance robust clinical evidence with healthcare resource constraints.

Addressing complex co-occurring conditions, Buhmann et al. investigate trauma-focused cognitive behavioral therapy (TF-CBT) adapted specifically for patients experiencing both PTSD and psychosis. They highlight the complexity and variability encountered in treating PTSD in the context of psychosis, including practical challenges such as treatment engagement, tolerability, and the necessity for individualized treatment modules. Their findings have profound implications for community mental health settings, underscoring the need for tailored clinical training, strategic resource allocation, and flexible implementation frameworks capable of addressing highly variable psychopathology in real-world contexts.

Together, these articles illustrate complementary pathways toward enhancing PTSD care through personalized approaches. Macy et al.'s digital therapeutic solution emphasizes real-time physiological data to enhance accessibility and reduce stigma. Guo et al.'s neuroscientific distinction between fear memory erasure and extinction underscores the ethical complexities of targeted neurobiological interventions. Shannon and Geller highlight MDMA-assisted psychotherapy's regulatory advancements, proposing integrated psychotherapeutic models beneficial to broader mental health conditions and marginalized populations. Buhmann et al. emphasize the critical role of adapting treatments to address the real-world complexities of co-occurring PTSD and psychosis, reinforcing the necessity of flexible clinical strategies.

These studies collectively advocate for policy support, international collaboration, and sustained investment to ensure that innovative, personalized treatments become accessible realities. Clinicians should leverage measurement-based approaches to personalize care, researchers should prioritize interdisciplinary validation studies, and policymakers must support flexible regulatory frameworks and funding strategies. By committing to these coordinated actions, the mental healthcare community can foster meaningful recovery, deeper understanding, and renewed hope for individuals affected by PTSD globally.

